# Diagnostic performance of Midkine ratios in fine-needle aspirates for evaluation of Cytologically indeterminate thyroid nodules

**DOI:** 10.1186/s13000-021-01150-y

**Published:** 2021-10-25

**Authors:** Le Zhou, Jinxi Jiang, Yantao Fu, Daqi Zhang, Tong Li, Qingfeng Fu, Chao Yan, Yifan Zhong, Gianlorenzo Dionigi, Nan Liang, Hui Sun

**Affiliations:** 1 Division of Thyroid Surgery, Jilin Provincial Key Laboratory of Surgical Translational Medicine, Jilin Provincial Precision Medicine Laboratory of Molecular Biology and Translational Medicine on Differentiated Thyroid Carcinoma, The China-Japan Union Hospital of Jilin University, 126 Xiantai Street, Changchun City, 130033 Jilin Province China; 2 Division of General and Endocrine Surgery, Istituto Auxologico Italiano IRCCS, Department of Medical Biotechnology and Translational Medicine, University of Milan, Milan, Italy; 3 Division Of Laboratory Medicine Center, The China-Japan Union Hospital of Jilin University, Changchun City, Jilin Province China

**Keywords:** Fine-needle aspiration, Thyroid cancer, Thyroid nodule, Midkine, Multinodular goiter

## Abstract

**Background:**

Fine-needle aspiration cytology (FNAC) is a basic diagnostic tool for thyroid nodules. However, 15–30% of nodules are cytologically indeterminate. Midkine (MK), a pleiotropic growth factor, is often upregulated in patients with cancers. This study aimed to evaluate the role of MK and its ratios in fine-needle aspirates (FNA) for predicting thyroid malignancy.

**Methods:**

This retrospective study included patients with thyroid nodules who underwent preoperative FNA and/or thyroidectomy between April 2017 and September 2017. MK levels in FNA washout were measured by enzyme-linked immunosorbent assay, and thyroglobulin (TG) and free thyroxine (FT4) levels in FNA washout were measured by chemiluminescent immunometric assays.

**Results:**

A total of 217 patients with 242 nodules were included in this study. The concentrations of TG, FT4, MK/TG, MK/FT4, and FT4/MK were significantly different between papillary thyroid carcinomas and benign thyroid nodules. Both MK/TG and MK/FT4 ratios were positively correlated with maximum tumor diameter, extrathyroidal extension, and T and N stages. The area under the curve for MK/TG was 0.719 with a cutoff value of 55.57 ng/mg, while the area under the curve for MK/FT4 was 0.677 with a cutoff value of 0.11 μg/pmol. FNAC in combination with MK/FT4 had a higher sensitivity (95% vs. 91%) and accuracy (96% vs. 92%) than FNAC alone for cytologically indeterminate specimens, those of unknown significance, or those suspected of malignancy.

**Conclusions:**

MK/FT4 and MK/TG may have diagnostic utility for evaluation of papillary thyroid carcinomas, particularly for cytologically indeterminate thyroid nodules.

**Supplementary Information:**

The online version contains supplementary material available at 10.1186/s13000-021-01150-y.

## Background

The incidence of thyroid nodules is high, with a reported prevalence of 19 to 67% in the general population [1]. Neck ultrasound (US) has well-known utility for evaluating thyroid nodules and is recommended as a first-line imaging approach to determine the need for further cytological examination [2]. US-guided fine-needle aspiration cytology (FNAC) is a minimally invasive procedure that can provide valuable clinical and pathological information through evaluation of thyroid nodule aspirates using the Bethesda classification system [3]. For most patients, the combination of US and FNAC is the optimal diagnostic approach. However, the reported proportion of specimens determined to be cytologically indeterminate using FNAC ranges from 15 to 30% [4]. Therefore, additional morphological and functional methods to complement these evaluations should be identified. Researchers have focused on US elastography and biomarkers, such as BRAF, RAS, RET/PTC, galactine-3, HBME-1, and cytokeratin 19, with the hope of making up for the defects of the existing technology [2, 5–7]. Since the measurement of thyroglobulin (TG) in FNA washout was first proposed as a supplementary method to FNAC for the detection of cervical lymph nodes metastases in 1992, growing numbers of studies have focused on the diagnostic performance of markers in FNA washout in thyroid cancer [8–11]. Despite the contribution made by these methods in aiding the diagnosis of uncertain nodules, they have not completely solved this problem. More reliable biomarkers need to be further explored and discovered, which will help clinical decision-making.

Midkine (MK), a 13-kDa pleiotropic growth factor, is often upregulated in patients with cancer [3, 12–15]. Upregulation of MK has been shown to be closely associated with several oncogenic characteristics, including increased cell proliferation, invasion, migration, and angiogenesis. Expression of MK, which is downstream of BRAF, is highly related to BRAF mutations [13]. In addition, higher expression of MK in patients with thyroid cancer has been associated with extrathyroidal invasion, lymph node metastases, and advanced tumor stage [2, 16]. Unfortunately, given its widespread expression in many cancer types, serum MK levels have relatively low diagnostic specificity for identifying thyroid cancer; however, determination of MK expression within nodules has been shown to have some diagnostic promise [4, 16]. Several studies have determined MK concentrations in FNA washout samples of thyroid nodules [4]. The MK-to-TG ratio (MK/TG) has been shown to have better diagnostic performance than the MK level alone [4]; however, the TG concentration in thyroid nodules is generally beyond the range of clinical detection (> 500 ng/mL), which can lead to an inaccurate or incalculable ratio. Since free thyroxine (FT4) is thyroid tissue-specific and expressed at the same order of magnitude as MK [17], we propose the use of the MK-to-FT4 ratio (MK/FT4) in this study, aiming to facilitating the diagnostic utility of MK concentrations.

In this study, we aimed to determine the diagnostic accuracy of MK concentrations, especially MK/FT4 and MK/TG, obtained from FNA in predicting malignancy in patients with thyroid nodules. We also aimed to examine the relationship of these variables with clinicopathologic characteristics, American College of Radiology Thyroid Imaging Reporting and Data System (ACR TI-RADS) categorization, and Bethesda categorization of FNAs.

## Materials and methods

### Study population

**Fig. 1:**
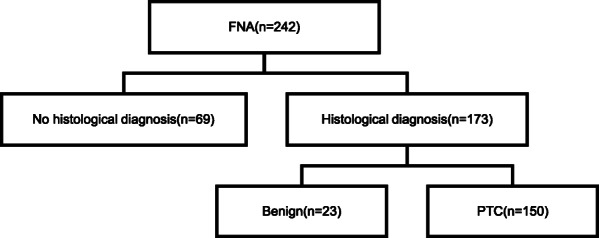
Flowchart of thyroid nodule evaluations. FNA, fine-needle aspiration; PTC, papillary thyroid carcinoma

Our subjects (*n* = 217) were consecutive patients with thyroid nodules who underwent preoperative FNA and/or thyroidectomy at the Division of Thyroid Surgery at China-Japan Union Hospital of Jilin University between April 2017 and September 2017. Patients were excluded if they had other thyroid diseases (e.g., Graves’ hyperthyroidism or Hashimoto’s thyroiditis), other malignant tumors, or liver, kidney, or nervous system diseases. Sequential thyroid nodule sample evaluation steps are shown in Fig. 1.

### Instruments and methods

#### Sonography

Thyroid US was performed preoperatively by two radiologists using an 8- to 13-MHz linear array probe (S50 PRO, Sonoscape, Shenzhen, China). Sonographic features considered suspicious for malignancy are presented in Additional file 1. Using thyroid US, nodules were classified from TR1 through TR5 based on the ACR TI-RADS.

#### Cytological examination

All preoperative FNAs were performed by two experienced surgeons (> 5 years of experience) under US guidance with local anesthesia. For each nodule, one to two FNA samples were obtained using a 22-G needle without applying suction. After the content of each needle was expelled onto a microscope slide for conventional cytology, the needle was rinsed with 600 μL of phosphate-buffered solution. The washout was then aliquoted and stored immediately at − 80 °C until assays were performed. Slides were stained using hematoxylin and eosin and evaluated by pathologists blinded to the original classification. Cytological evaluations were classified into categories I through VI using the Bethesda System for Reporting Thyroid Cytopathology [18] as follows: I, non-diagnostic or unsatisfactory; II, benign; III, atypia of undetermined significance or follicular lesion of undetermined significance; IV, follicular neoplasm or suspicious for a follicular neoplasm; V, suspicious for malignancy; or VI, malignancy.

#### MK measurement

FNA washout samples were transported to the laboratory in a temperature-controlled system. MK measurements were performed using a human MK ELISA Kit (E-EL-H2297c, Elabscience, Houston, TX) with modifications. The detection range of this kit has been shown to be 0.156–10 ng/mL, with normal serum MK levels generally less than 0.5 ng/mL [19]. This kit can detect natural or recombinant human MK without obvious cross-reactivity with other related proteins and with in-plate and inter-plate coefficients of variation of < 10%.

#### TG and FT4 measurements

TG and FT4 levels were measured in FNAs. TG and FT4 were detected by chemiluminescent immunometric assays (cat. Nos. 06445896 for TG and 07976836 for FT4, Roche Diagnostics GmbH, Mannheim, Germany). For TG detection, the washout was diluted 10:1000.

#### Histopathology

Thyroid nodules corresponding to the FNA underwent diagnostic evaluations of surgical histopathology by an expert panel blinded to all MK data. All nodules were classified as either benign thyroid nodules or papillary thyroid carcinomas (PTC)s.

### Statistical analysis

Data were analyzed using SPSS 22.0 (IBM, Armonk, NY) and presented as mean ± standard deviation, median (range), or number (percentage). Kruskal–Wallis and Mann–Whitney *U* tests were used for multiple comparisons. Spearman bivariate correlations were made among the variables and clinicopathological characteristics. Multivariate linear regression analysis was used to screen for independent significant risk factors. Receiver operating characteristic (ROC) curves were used to calculate areas under the ROC curve (AUCs) and to evaluate diagnostic values. *P*-values less than 0.05 were considered to indicate statistical significance.

## Results

### Baseline demographic and clinical characteristics of study cohort

As shown in Table 1, a total of 217 patients (43 males and 174 females**)** with 242 nodules (23 benign thyroid nodules and 150 PTCs) determined by histopathological analysis were evaluated. Most nodules were categorized as either FNA II (28.1%) or FNA VI (58.3%) on cytologic analysis. Nodules were classified into the five ACR TI-RADS categories (TR1-TR5) as follows: 3.6, 4.1, 5.9, 29.5, and 56.8%, respectively.

**Table 1:** Baseline demographic and clinical characteristics of study cohort

Demographic and clinical characteristics	Total
Number of patients	217
Gender	
Male	43 (19.8%)
Female	174 (80.2%)
Age (years)	
Children (≤18)	0 (0%)
Youth (19–44)	100 (46.1%)
Middle age (45–59)	103 (47.5%)
Elderly (≥60)	14 (6.5%)
Number of nodules	242
Histopathology	
Absent	69 (28.5%)
Benign	23 (9.5%)
PTC	150 (62.0%)
^a^FNA	
I	8 (3.3%)
II	68 (28.1%)
III	4 (1.7%)
IV	0 (0%)
V	21 (8.7%)
VI	141 (58.3%)

^a^ *FNA* fine-needle aspiration

### MK ratios in benign and malignant thyroid nodules

**Fig. 2:**
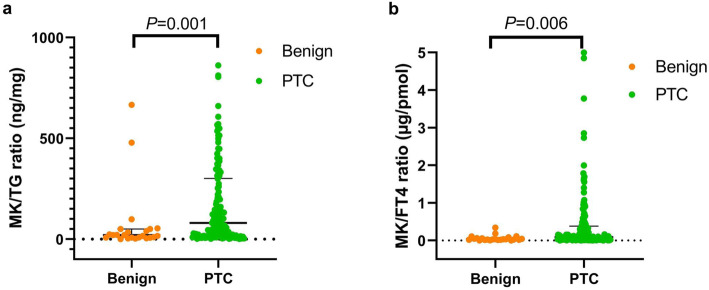
Scatter plots of (**a**) midkine (MK)/thyroglobulin (TG) ratio and (**b**) MK/free thyroxine (FT4) ratio in patients with benign thyroid nodules versus papillary thyroid carcinomas (PTCs) confirmed by histopathology. Both MK/TG and MK/FT4 ratios were significantly higher for PTCs than for benign thyroid nodules (*P* < 0.05)

We first compared MK, FT4, and TG concentrations and MK ratios (MK/TG, MK/FT4, and FT4/MK) between nodules determined to be benign and PTCs by histopathological analysis. As illustrated in Table 2 and Fig. 2, five of the indicators (with the exception of MK level), had significantly different values between benign and malignant nodules (*P* < 0.05). MK/TG was significantly higher in PTCs (80.59 ng/mg) than benign nodules (20.16 ng/mg) (*P* = 0.001). Similarly, MK/FT4 was significantly higher in PTCs (0.09 μg/pmol) than benign nodules (0.03 μg/pmol) (*P* = 0.006). Finally, TG, FT4 and FT4/MK were significantly higher in benign nodules than PTCs. These findings suggested that MK ratios may have utility in distinguishing benign nodules from PTCs.

**Table 2:** MK ratios in benign versus malignant thyroid nodules

Indicators	Benign	PTC	***P*** value
**MK (ng/ml)**	0.31 (0.02,3.83)	0.41 (0.01,16.93)	0.402
**TG (ng/ml)**	24,375.00 (336.50,300,500.00)	5467.00 (155.00,3,224,500.00)	0.008*
**MK/TG (ng/mg)**	20.16 (0.31.666.65)	80.59 (1.52,4021.37)	0.001*
**FT4 (pmol/L)**	9.54 (1.04,332.90)	3.87 (0.33,141.30)	0.037*
**MK/FT4(μg/pmol)**	0.03 (0.002,0.34)	0.09 (0.001,8.21)	0.006*
**FT4/MK (pmol/μg)**	32.48 (2.94,453.94)	11.50 (0.12,910.94)	0.006*

* *P*<0.05

### Relationship between MK ratios and clinicopathological features

We further explored the associations of MK, TG, FT4, and MK ratios with several clinicopathological features with prognostic implications, including the number of nodules, maximum tumor diameter (MTD), extrathyroidal extension (ETE), and TNM staging. As shown in Table 3, MK expression significantly differed according to MTD (*P* = 0.000), T stage (*P* = 0.008), and N stage (*P* = 0.006). TG and FT4 levels, however, only differed according to ETE. Both MK/TG and MK/FT4 increased with MTD (*P* = 0.009 and 0.001, respectively) and significantly differed according to ETE (*P* = 0.001 and 0.003, respectively). MK/TG and MK/FT4 increased with T stage (both *P* = 0.002). However, only MK/FT4 increased with N stage (*P* = 0.025). There were also significant differences for FT4/MK in MTD (*P* = 0.001), ETE (*P* = 0.003), T stage (*P* = 0.002), and N stage (*P* = 0.025). Both MK/TG and MK/FT4 were positively correlated with MTD, ETE, T stage, and N stage despite correlation coefficients less than 0.5 (*P* < 0.05; Table 4). Furthermore, multivariate linear regression analyses were carried out based on the above results, but data were not shown due to the lack of fitted models. These findings suggest that MK/TG and MK/FT4 may have potential prognostic utility for PTC.

**Table 3:** MK ratios in different clinicopathological features

	MK (ng/ml)	***P*** value	TG (ng/ml)	***P*** value	MK/TG (ng/mg)	***P*** value	FT4(pmol/L)	***P*** value	MK/FT4(μg/pmol)	***P*** value	FT4/MK (pmol /μg)	***P*** value
**Number of nodules**		0.392		0.431		0.158		0.449		0.324		0.324
Solitary	0.47 (0.02,16.93)		5003.25 (155,230,050)		96 (2.10,4021.67)		3.8 (0.33,78.57)		0.1 (0.002,6.84)		9.57 (0.15,563.76)	
Multiple	0.39 (0.02,12.07)		5711.25 (560,3,224,500)		70.97 (1.52,1202.08)		4.06 (0.48,141.3)		0.07 (0.001,8.21)		14.81 (0.12,910.94)	
^**a**^**MTD**		0.000*		0.691		0.009*		0.395		0.001*		0.001*
≤ 1 cm	0.27 (0.013,12.07)		5711.29 (171,230,050)		58.83 (1.52,4021.37)		4.05 (0.54,96.95)		0.06 (0.001,8.21)		17.06 (0.12,910.94)	
>1 cm, ≤ 2 cm	0.56 (0.06,16.93)		4119.5 (155,178,100)		161.09 (3.56,2380.22)		3.85 (0.33,80.69)		0.32 (0.004,6.32)		3.16 (0.16,222.71)	
>2 cm, ≤4 cm	2.50 ± 2.84		8205 (199,3,224,500)		389.81 ± 516.56		2.29 (0.39,141.3)		1.07 ± 1.47		9.53 ± 16.76	
^**b**^**ETE**		0.241		0.009*		0.001*		0.009*		0.003*		0.003*
No invasion	0.37 (0.015,16.93)		9230 (1845,300,500)		40.31 (0.31,1766.59)		6.56 (0.39,332.9)		0.05 (0.001,8.21)		21.92 (0.12,708.99)	
^c^Capsule and the outside	0.55 (0.02,9.79)		3457.25 (155,3,224,500)		138.95 (2.37,4021.37)		2.73 (0.33,141.3)		0.21 (0.005,6.84)		4.74 (0.15,222.71)	
^d^Soft tissues or strap muscles	0.35 (0.013,6.42)		3479.75 (1117.519935)		210.42 (3.99,861.67)		5.2 (0.51,96.96)		0.0096 (0.001,6.32)		15.66 (0.16,910.94)	
**T stage**		0.008*		0.119		0.002*		0.269		0.002*		0.002*
T1a	0.27 (0.02,12.07)		5807.5 (171,230,050)		60.50 (1.52,4021.37)		3.96 (0.54,89.70)		0.06 (0.001,8.21)		16.63 (0.12,708.99)	
T1b	0.61 (0.06,16.93)		5003.25 (155,178,100)		137.38 (3.56,2380.22)		4.08 (0.33,80.69)		0.31 (0.005,2.85)		3.24 (0.35,222.71)	
T2	1.37 (0.30,8.05)		8205 (199,3,224,500)		196.78 (2.37,1766.59)		2.29 (0.39,141.3)		0.59 (0.02,5.00)		1.68 (0.20,56.72)	
T3b	0.35 (0.11,3.22)		3327.25 (1117.519935)		210.42 (8.82,861.67)		3.05 (0.51,96.96)		0.10 (0.002,6.32)		15.66 (0.16,551.37)	
T4a	3.22 (0.01,6.42)		6490.5 (3255,9726)		332.18 (3.99,660.37)		9.29 (6.73,11.84)		0.48 (0.001,0.95)		455.99 (1.05,910.94)	
**N stage**		0.006*		0.635		0.064		0.587		0.025*		0.025*
N0	0.26 (0.02,9.79)		4645.00 (171.00,230,050.00)		46.35 (1.52,4021.37)		4.53 (0.33,78.50)		0.07 (0.001,6.84)		15.16 (0.15,708.99)	
N1	0.52 (0.013,16.93)		6162.50 (155.00,3,224,500.00)		111.80 (2.37,2380.22)		3.72 (0.39,141.30)		0.13 (0.001,8.21)		7.94 (0.12,910.94)	

^a^ *MTD* maximum tumor diameter, ^b^ *ETE* extrathyroidal extension, ^c^ *capsule and the outside* invasion through the capsule and reached the outside, ^d^ *soft tissues or strap muscles* perithyroidal soft tissues or strap muscles

*P<0.05

**Table 4:** Correlation between MK ratios and clinicopathological features

Clinicopathology	MK		TG		MK/TG		FT4		MK/FT4		FT4/MK	
	r	***p***	r	***p***	r	***p***	r	***p***	r	***p***	r	***p***
**Number of nodules**	−0.070	0.394	0.065	0.433	−0.116	0.158	0.062	0.451	−0.081	0.325	0.081	0.325
^a^**MTD**	0.352	0.000*	−0.105	0.199	0.359	0.000*	−0.180	0.027*	0.374	0.000*	−0.374	0.000*
^b^**ETE**	0.105	0.170	−0.235	0.002*	0.278	0.000*	−0.215	0.004*	0.235	0.002*	−0.235	0.002*
**T stage**	0.221	0.003*	−0.164	0.031*	0.319	0.000*	−0.151	0.048*	0.303	0.000*	−0.303	0.000*
**N stage**	0.254	0.002*	0.027	0.742	0.166	0.042*	−0.046	0.578	0.193	0.018*	−0.193	0.018*

^a^ *MTD* maximum tumor diameter, ^b^ *ETE* extrathyroidal extension

*P<0.05

### Diagnostic utility of MK ratios for identifying malignant thyroid nodules

**Fig. 3:**
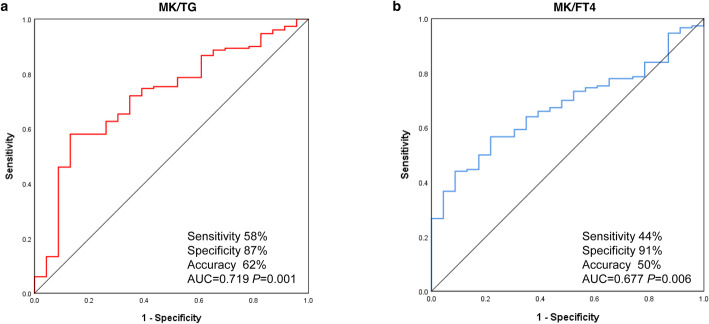
Diagnostic value of midkine (MK)/thyroglobulin (TG) and MK/free thyroxine (FT4) ratios for papillary thyroid carcinoma (PTC). Receiver operating characteristic curves were drawn to assess the diagnostic utility of the (**a**) MK/TG ratio and the (**b**) MK/FT4 ratio in preoperatively distinguishing PTCs from benign thyroid nodules. The areas under the curve (AUC) for the MK/TG and MK/FT4 ratios were 0.719 (*P* = 0.001) and 0.677 (*P* = 0.006), respectively, with no significant difference (*P* = 0.896). The cut-off values of MK/TG and MK/FT4 were 55.57 ng/mg (sensitivity 58%, specificity 87%, accuracy 62%) and 0.11 μg/pmol (sensitivity 44%, specificity 91%, accuracy 50%), respectively

To evaluate the diagnostic utility of the six indicators for distinguishing PTCs from benign nodules, we drew ROC curves and analyzed AUCs. As illustrated in Fig. 3, only MK/TG and MK/FT4 showed significant AUC values greater than 0.5. The AUCs and optimal cutoffs were 0.719 and 55.57 ng/mg for MK/TG and 0.677 and 0.11 μg/pmol for MK/FT4, respectively. No significant difference was observed between MK/TG and MK/FT4. Although MK/TG showed a higher sensitivity (58%) and accuracy (62%) than MK/FT4 (44 and 50%, respectively), MK/FT4 showed a better specificity (91%) than MK/TG (87%) (Additional file 1). These findings suggest that both ratios have good diagnostic performance for discriminating benign nodules from PTCs.

### Performance of MK ratios in combination with FNAC classification

We further explored whether MK ratios could complement preoperative FNAC-based diagnoses. As shown in Additional file 2, the six indicators had significantly different values for the five FNA categories (*P* < 0.05); however, these observed differences were mainly accounted for by FNA categories II and VI (Table 5), a finding that is consistent with our clinical experience. Since FNAC is known to have limitations for certain tumor types, we placed more emphasis on FNA categories I, III, and V. FNAC combined with MK/FT4 had higher sensitivity (95%) and accuracy (96%) than FNAC alone (91 and 92%, respectively) (Table 6). FNAC in combination with MK/TG did not change the diagnostic performance significantly, demonstrating that MK/FT4 had better diagnostic utility for identifying malignant nodules in combination with FNAC, particularly for FNA categories I, III, and V.

**Table 5:** The expression levels of MK ratios in FNA categories II and VI

Indicators	FNA II	FNAVI	***P*** value
**MK (ng/ml)**	0.23 (0.01,6.14)	0.42 (0.01,16.93)	0.005*
**TG (ng/ml)**	16,210.00 (147.50,552,650.00)	4831.50 (155.00,3,224,500.00)	0.000*
**MK/TG (ng/mg)**	16.82 (0.31,1752.56)	100.15 (1.52,4021.37)	0.000*
**FT4 (pmol/L)**	9.32 (0.42,332.90)	3.63 (0.33,141.30)	0.000*
**MK/FT4(μg/pmol)**	0.02 (0.001,0.62)	0.11 (0.01,8.21)	0.000*
**FT4/MK (pmol/μg)**	44.60 (1.61,1527.88)	9.37 (0.12,910.94)	0.000*

*P<0.05

**Table 6:** Diagnostic utility comparison of MK ratios in combination with FNAC

	Sensitivity (%)	Specificity (%)	^**a**^PPV (%)	^**b**^NPV (%)	Accuracy (%)
**FNA**	91	100	100	67	92
**MK/TG (ng/mg)**	36	100	100	22	46
**MK/FT4 (μg/pmol)**	18	100	100	18	31
**FNA + MK/TG (ng/mg)**	91	100	100	67	92
**FNA + MK/FT4 (μg/pmol)**	95	100	100	80	96

^a^ *PPV* positive predictive value, ^b^ *NPV* negative predictive value

Similarly, we evaluated the diagnostic utility of these indicators when combined with the ACR TI-RADS. Based on sonographic features considered suspicious for malignancy (Additional file 3) and the histopathological diagnosis, the ROC curve for ACR TI-RADS had an AUC of 0.848 (*P* = 0.001) with an optimal cutoff value of 4.5 points (Additional file 4). Based on this finding, we compared the diagnostic efficacy of combining ACR TI-RADS with MK ratios. As shown in Additional file 5, higher sensitivities were identified when combining ACR TI-RADS with either MK/FT4 (91%) or MK/TG (89%) than when using ACR TI-RADS alone (83%). Higher accuracies were also identified when combining ACR TI-RADS with either MK/FT4 (88%) or MK/TG (87%) than ACR TI-RADS alone (83%). Lower specificities were found, however, when combining ACR TI-RADS with either MK/FT4 (65%) or MK/TG (70%) than ACR TI-RADS alone (74%). These findings suggest that both MK ratios can complement preoperative sonographic diagnoses.

## Discussion

Recently, thyroid nodules have become a globally prevalent endocrine disease. With the continuous development of diagnostic techniques such as US and FNA, the proportion of malignant thyroid nodules has been increasing annually [20]. At present, even though FNAC is known to have a high preoperative diagnosis rate, there are still unavoidable false-negative results. In order to improve the diagnostic efficiency of indeterminate thyroid nodules, studies have focused on integrating cytological information with clinical and US risk factors of thyroid malignancy [21]. Furthermore, several other attempts to employ novel biomarkers have been made to solve these problems.

MK, as a novel and cost-effective biomarker, has been shown to have high expression in at least 20 different types of cancer, including thyroid cancer [22–26]. Elevated MK in thyroid tissue has been reported to be highly related to the presence of PTC [2, 12, 27, 28]. Kato et al. found that MK was undetectable in normal thyroid tissue; however, the positivity rate and staining intensity on immunohistochemical assays were significantly increased in PTC tissue [12]. In addition, the grade of immunohistochemical staining for MK in thyroid tissue has been used to identify PTCs and multinodular goiters, as well as to predict the presence of metastases [28]. Other studies have demonstrated that MK concentrations in thyroid tissue are elevated in patients with PTCs compared to benign thyroid nodules, which can preoperatively predict tumorigenesis in highly suspicious thyroid nodules [4, 16]. The role of MK in tumorigenesis may be related to its effects on cancer cell proliferation, cell survival, apoptosis, and epithelial-mesenchymal transitions [29–31].

On the other hand, the relationship between serum MK levels and malignancy is not as clear. While Jee et al. identified a significant difference in serum MK concentrations between patients with PTCs and benign nodules, Shao et al. found conflicting results [2, 4]. Therefore, it is possible that serum MK levels may have utility for differentiating benign thyroid nodules from PTC; however, these evaluations are likely to have a relatively lower sensitivity, specificity, and accuracy [32, 33]. Markedly enhanced MK expression levels in patients with various pathological conditions, especially cancers, inevitably leads to low diagnostic specificity [22]. For this reason, we first investigated MK/FT4 in FNA washout from thyroid nodules to assess its potential to complement current preoperative diagnostic strategies. Compared with MK/TG, MK/FT4 is more convenient and feasible.

In our study, we found that both MK/FT4 and MK/TG may be equally useful as biomarkers for quantitative diagnoses of PTC, particularly for cytologically indeterminate thyroid nodules. Furthermore, although we did not identify a significant relationship between MK levels and extrathyroidal invasion, MK levels were significantly associated with T and N staging, which is consistent with findings of previous studies [2, 16, 34]. We also found that MK/TG and MK/FT4 were closely associated with MTD, ETE, T stage, and N stage. These findings further strengthen the importance of these ratios as potential biomarkers, demonstrating they may play critical roles in predicting PTC risk.

The success of preoperative diagnostic evaluations using FNAC and US examinations mainly depends on the experience of pathologists and radiologists and the level of care offered by hospitals. Fortunately, the ratio method proposed in this study may reduce the relative impact of operator-dependent FNAC and US. This study demonstrated that FNAC combined with MK/FT4 had superior diagnostic performance than FNAC alone for nodules classified as either FNA I, III or V. In addition, US in combination with either MK/TG or MK/FT4 had an improved preoperative diagnostic performance than US alone. Therefore, MK/FT4 may be a better quantitative biomarker than MK/TG and may have utility as a complementary cytological diagnostic tool, a finding that certainly should be further explored in future studies.

Finally, this study has some limitations. First, there were significant efforts to improve the diagnostic work-up of indeterminate thyroid nodules, including the use of US classification systems, the combination of US and cytological features, and the use of elastography. Novel biomarkers should be competitive in terms of cost-effectivenes with currently available procedures. Secondly, the cost and feasability of MK measurement in FNAs is a necessary issue to analyze in the future.

## Supplementary Information

**Additional file 1.** Diagnostic utility of midkine ratios for identifying malignant thyroid nodules.

**Additional file 2.** The expression levels of midkine ratios in different fine-needle aspirate categories.

**Additional file 3.** Sonographic features considered suspicious.

**Additional file 4. **Diagnostic ability of the American College of Radiology Thyroid Imaging Reporting and Data System (ACR TI-RADS) for distinguishing papillary thyroid carcinomas (PTCs) from benign nodules. Receiver operating characteristic curves were drawn based on the cumulative score of five sonographic features for assessing the diagnostic capability of ACR TI-RADS. The area under the curve (AUC) of ACR TI-RADS was 0.848 (*P* = 0.001) with a cut-off value of 4.5 points.

**Additional file 5:.** Diagnostic utility comparison of midkine ratios in combination with the American College of Radiology Thyroid Imaging Reporting and Data System.

## Data Availability

The datasets used and/or analyzed during the current study are available from the corresponding author on reasonable request.

## Abbreviations

AUC: Area under curve
BRAF: V-raf murine sarcoma viral oncogene homolog B1
ETE: Extrathyroidal extension
FNAC: Fine-needle aspiration cytology
FT4: Free thyroxine
MK: Midkine
MTD: Maximum tumor diameter
NPV: Negative predictive value
PPV: Positive predictive value
ROC: Receiver operating characteristic curve
Tg: Thyroglobulin
US: Ultrasound
