# Supplementary material for: Diagnostic performance of Midkine ratios in fine-needle aspirates for evaluation of Cytologically indeterminate thyroid nodules
Source: Diagn Pathol. 2021 Oct 25;16:92. doi: 10.1186/s13000-021-01150-y (PMC8543763; doi:10.1186/s13000-021-01150-y)
Supplement: Supplementary file 1 — Additional file 1. Diagnostic utility of midkine ratios for identifying malignant thyroid nodules. [file 13000_2021_1150_MOESM1_ESM.docx]

**Additional file 1 Diagnostic utility of MK ratios for identifying malignant thyroid nodules**

|  | **^a^AUC** | **Optimal cut-off value** | **Sensitivity (%)** | **Specificity (%)** | **^b^PPV (%)** | **^c^NPV (%)** | **Accuracy (%)** | **Youden Index** | ***P* value** |
| --- | --- | --- | --- | --- | --- | --- | --- | --- | --- |
| **MK/TG** | 0.719 | 55.57(ng/mg) | 58 | 87 | 97 | 24 | 62 | 0.45 | 0.001* |
| **MK/FT4** | 0.677 | 0.11(µg/pmol) | 44 | 91 | 97 | 20 | 50 | 0.353 | 0.006* |

^a^ AUC=area under the curve; ^b^ PPV=positive predictive value; ^c^ NPV=negative predictive value

**P*＜0.05
