# Supplementary material for: Diagnostic performance of Midkine ratios in fine-needle aspirates for evaluation of Cytologically indeterminate thyroid nodules
Source: Diagn Pathol. 2021 Oct 25;16:92. doi: 10.1186/s13000-021-01150-y (PMC8543763; doi:10.1186/s13000-021-01150-y)
Supplement: Supplementary file 2 — Additional file 2. The expression levels of midkine ratios in different fine-needle aspirate categories. [file 13000_2021_1150_MOESM2_ESM.docx]

**Additional file 2 The expression levels of MK ratios in different FNA categories**

| **Indicators** | **FNA I** | **FNA II** | **FNA III** | **FNA V** | **FNA VI** | ***P* value** |
| --- | --- | --- | --- | --- | --- | --- |
| **MK (ng/ml)** | 0.21±0.12 | 0.23(0.01,6.14) | 0.39(0.17,0.45) | 0.35(0.07,4.09) | 0.42(0.01,16.93) | 0.021* |
| **TG (ng/ml)** | 22403.31±23340.78 | 16210.00(147.50,552650.00) | 6762.50(5615.00,14445.00) | 9230.00(560.00,230050.00) | 4831.50(155.00,3224500.00) | 0.004* |
| **MK/TG (ng/mg)** | 13.47(2.96,143.49) | 16.82(0.31,1752.56) | 39.85(26.40,80.32) | 33.10(5.22,498.34) | 100.15(1.52,4021.37) | 0.000* |
| **FT4 (pmol/L)** | 5.90(1.11,36.72) | 9.32(0.42,332.90) | 4.26(2.07,6.79) | 6.56(0.81,80.69) | 3.63(0.33,141.30) | 0.006* |
| **MK/FT4(µg/pmol)** | 0.04±0.02 | 0.02(0.001,0.62) | 0.09(0.06,0.11) | 0.03(0.01,1.79) | 0.11(0.01,8.21) | 0.000* |
| **FT4/MK (pmol/µg)** | 41.27±26.76 | 44.60(1.61,1527.88) | 11.74(9.00,17.80) | 30.34(0.56,151.80) | 9.37(0.12,910.94) | 0.000* |

**P*＜0.05
