# Supplementary material for: Diagnostic performance of Midkine ratios in fine-needle aspirates for evaluation of Cytologically indeterminate thyroid nodules
Source: Diagn Pathol. 2021 Oct 25;16:92. doi: 10.1186/s13000-021-01150-y (PMC8543763; doi:10.1186/s13000-021-01150-y)
Supplement: Supplementary file 3 — Additional file 3. Sonographic features considered suspicious. [file 13000_2021_1150_MOESM3_ESM.docx]

**Additional file 3 Sonographic features considered suspicious**

| Features | Appearance |
| --- | --- |
| **Composition** |  |
|  | Mixed cystic and solid |
|  | Solid or almost completely solid |
| **Echogenicity** |  |
|  | Hyperechoic or isoechoic |
|  | Hypoechoic |
|  | Very hypoechoic |
| **Shape** |  |
|  | Taller than wide |
| **Margin** |  |
|  | Lobulated/irregular |
|  | Extra-thyroidal extension |
| **Echogenic foci** |  |
|  | Macro-calcification |
|  | Peripheral/rim calcification |
|  | Punctate echogenic foci |
