# Supplementary figures and images for: Diagnostic performance of Midkine ratios in fine-needle aspirates for evaluation of Cytologically indeterminate thyroid nodules
Source: Diagn Pathol. 2021 Oct 25;16:92. doi: 10.1186/s13000-021-01150-y (PMC8543763; doi:10.1186/s13000-021-01150-y)

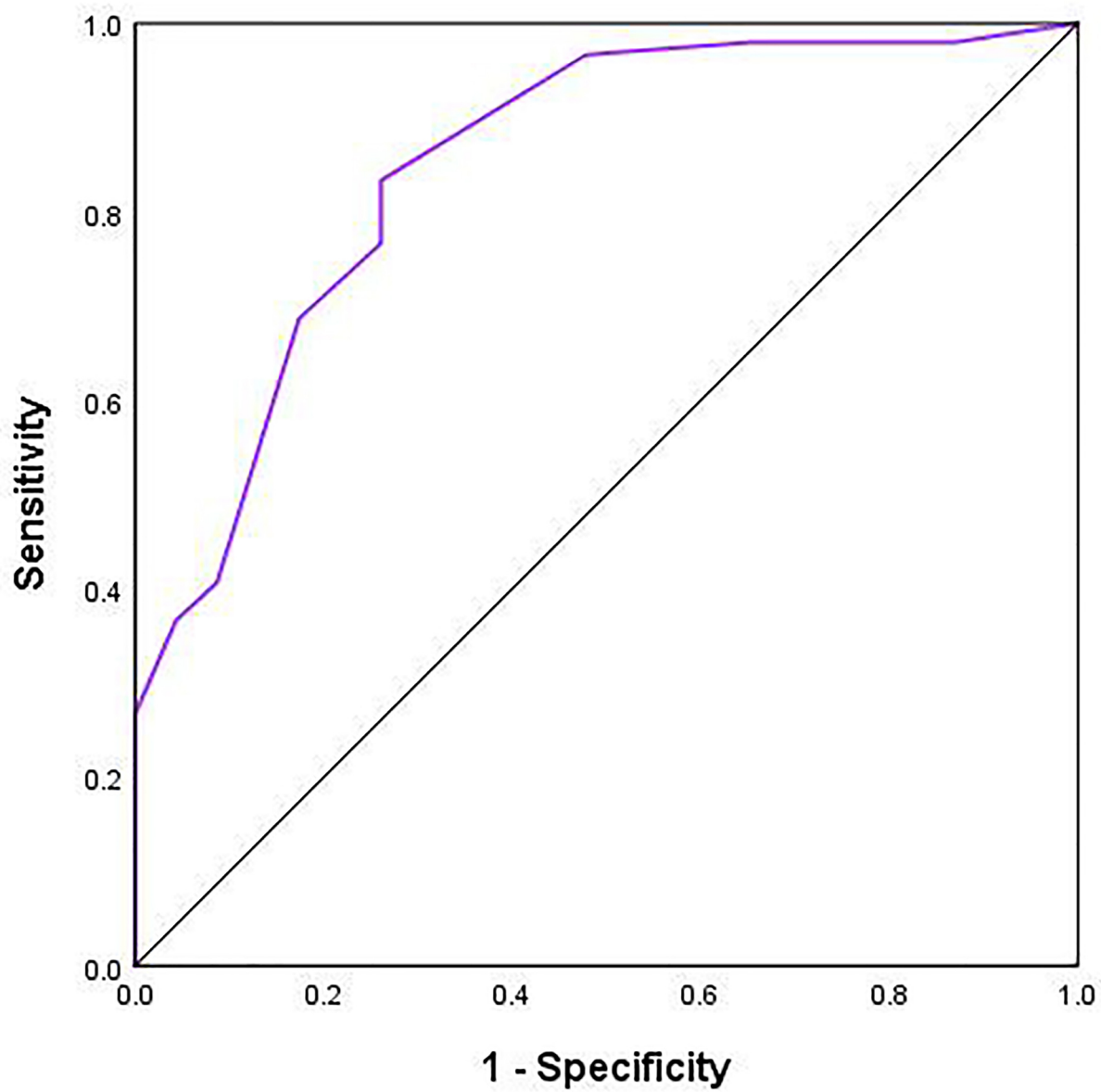

Supplement: Supplementary file 4 — Additional file 4. Diagnostic ability of the American College of Radiology Thyroid Imaging Reporting and Data System (ACR TI-RADS) for distinguishing papillary thyroid carcinomas (PTCs) from benign nodules. Receiver operating characteristic curves were drawn based on the cumulative score of five sonographic features for assessing the diagnostic capability of ACR TI-RADS. The area under the curve (AUC) of ACR TI-RADS was 0.848 (P = 0.001) with a cut-off value of 4.5 points. [file 13000_2021_1150_MOESM4_ESM.pdf]
