# Supplementary material for: Diagnostic performance of Midkine ratios in fine-needle aspirates for evaluation of Cytologically indeterminate thyroid nodules
Source: Diagn Pathol. 2021 Oct 25;16:92. doi: 10.1186/s13000-021-01150-y (PMC8543763; doi:10.1186/s13000-021-01150-y)
Supplement: Supplementary file 5 — Additional file 5:. Diagnostic utility comparison of midkine ratios in combination with the American College of Radiology Thyroid Imaging Reporting and Data System. [file 13000_2021_1150_MOESM5_ESM.docx]

**Additional file 5 Diagnostic utility comparison of MK ratios in combination with ACR TI-RADS**

|  | Sensitivity (%) | Specificity (%) | ^a^PPV (%) | ^b^NPV (%) | Accuracy (%) |
| --- | --- | --- | --- | --- | --- |
| **ACR TI-RADS** | 83 | 74 | 95 | 40 | 83 |
| **ACR TI-RADS+MK/TG (ng/mg)** | 91 | 65 | 94 | 54 | 88 |
| **ACR TI-RADS+MK/FT4 (µg/pmol)** | 89 | 70 | 95 | 50 | 87 |

a PPV=positive predictive value; b NPV=negative predictive value
